# Description of longitudinal tumor evolution in a case of multiply relapsed clear cell sarcoma of the kidney

**DOI:** 10.1002/cnr2.1458

**Published:** 2021-12-29

**Authors:** Tomoki Yaguchi, Shunsuke Kimura, Masahiro Sekiguchi, Yasuo Kubota, Masafumi Seki, Kenichi Yoshida, Yuichi Shiraishi, Keisuke Kataoka, Yoichi Fujii, Kentaro Watanabe, Mitsuteru Hiwatari, Satoru Miyano, Seishi Ogawa, Junko Takita

**Affiliations:** ^1^ Department of Pediatrics, Graduate School of Medicine The University of Tokyo Tokyo Japan; ^2^ Department of Pediatrics, Graduate School of Biomedical Sciences Hiroshima University Hiroshima Japan; ^3^ Department of Pathology and Tumor Biology, Graduate School of Medicine Kyoto University Kyoto Japan; ^4^ Division of Cellular Signaling National Cancer Center Research Institute Tokyo Japan; ^5^ Laboratory of DNA Information Analysis, Human Genome Center, Institute of Medical Science The University of Tokyo Tokyo Japan; ^6^ Department of Pediatrics, Graduate School of Medicine Kyoto University Kyoto Japan

**Keywords:** *BCOR*‐ITD, clear cell sarcoma of the kidney, clonal evolution, relapse

## Abstract

**Background:**

Clear cell sarcoma of the kidney (CCSK) is the second most common pediatric renal tumor.

**Case:**

A 2‐year‐old boy was diagnosed with CCSK, which relapsed four times until he yielded to the disease at the age of 7 years. To characterize the longitudinal genetic alterations occurring in the present case, we performed targeted‐capture sequencing by pediatric solid tumors panel (381 genes) for longitudinally sampled tumors, including autopsy samples of metastasis. Internal tandem duplication of *BCOR* (*BCOR*‐ITD) was the only truncal mutation, confirming the previously reported role of *BCOR*‐ITD in CCSK.

**Conclusion:**

Acquisition of additional mutations along tumor relapses and detection of metastasis‐specific mutations were reminiscent of the tumor progression and therapeutic resistance of this case, leading to clonal selection and a dismal fate.

## 1 INTRODUCTION

Clear cell sarcoma of the kidney (CCSK) makes up 4% of all primary renal malignancies in children.^1^ Although its prognosis has significantly improved recently,^1^, ^2^, ^3^, ^4^, ^5^ the relapse rate is still high and the prognosis of patients with relapses is extremely poor.^6^ Internal tandem duplication of *BCOR* (*BCOR*‐ITD),^7^, ^8^ *YWHAE‐NUTM2B/E* fusion,^9^, ^10^ and *BCOR*‐*CCNB3* fusion^11^ have been reported in CCSK cases; however, the genetic mechanisms associated with tumor recurrence and metastasis are still poorly understood. In this study, we assessed the genetic mechanisms of CCSK recurrence and metastasis by analyzing longitudinally sampled tumors extracted from a single case: specimens at diagnosis, each relapse, and autopsy.

## 2 CASE REPORT

**FIGURE 1:**
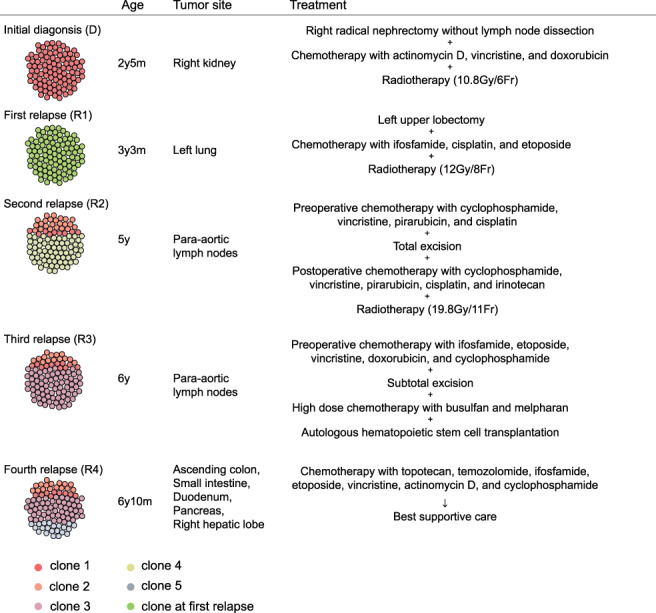
Clinical presentation in this case. Each mutation detected by targeted‐capture sequencing was classified based on the detected samples and variant allele frequency, while there were no data available for clone at first relapse. Estimated cell population at each sample is shown

A 2‐year‐old boy presented with a 2‐month history of abdominal distention. At the initial diagnosis, a tumor (17 × 14 × 11 cm) in the right kidney without metastasis (Stage‐I) was detected by enhanced computed tomography (CT) (Figure S1). The patient underwent a right radical nephrectomy without lymph node dissection (Figure 1), and pathological findings led to a diagnosis of CCSK. He received chemotherapy consisting of actinomycin D, vincristine, and doxorubicin (DD‐4A) with radiotherapy (10.8Gy/6Fr) and this combination therapy successfully induced complete remission. However, 4 months after the completion of treatment, a first relapse occurred at his left lung. He received six courses of chemotherapy consisting of ifosfamide, carboplatin, and etoposide (ICE) with radiotherapy (12Gy/8Fr) after a left upper lobectomy, and then achieved a second remission. At the age of 5 years, a second relapse developed at the para‐aortic lymph nodes. A total excision was done after three courses of chemotherapy consisting of cyclophosphamide, vincristine, pirarubicin, and cisplatin. Histologically, viable tumor cells remained with fibrosis and mucoid substances (Supplementary Material S1). Postoperative chemoradiotherapy induced a third remission. At the age of 6 years, he suffered from a third relapse at the para‐aortic lymph nodes, the same location as the second relapse. Chemotherapy consisting of ifosfamide, etoposide, vincristine, doxorubicin, and cyclophosphamide resulted in a poor therapeutic response. The patient underwent a subtotal excision of the lesion. Pathological examination of resected specimen detected viable tumor cells among therapy‐related necrosis and fibrosis (Supplementary material S1). High dose chemotherapy (busulfan and melpharan) with autologous hematopoietic stem cell transplantation (aHSCT) induced a fourth remission; however, 4 months later, a fourth relapse occurred at the ascending colon, small intestine, duodenum, pancreas, and right hepatic lobe, and he yielded to the advanced disease at the age of 7 years.

## 3 RESULTS

**FIGURE 2:**
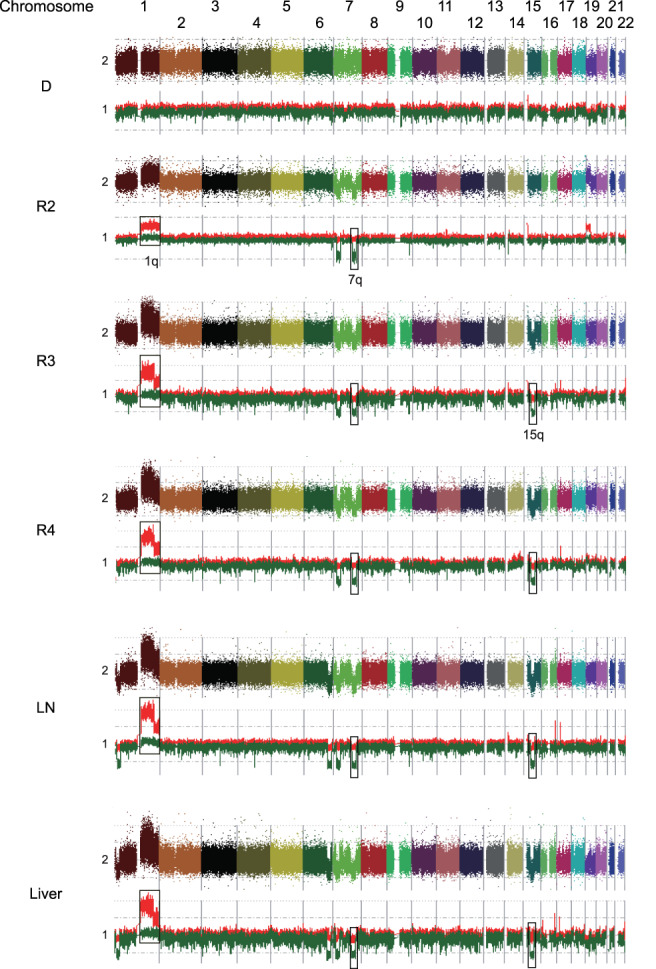
Results of SNP array analysis in each sample. Colorful dots at the top represent signal of each probe (raw data). Red and green lines at the bottom show allele‐specific copy number, respectively. Consistent copy number alterations on chromosomes 1q, 7q, and 15q were detected. D, initial diagnosis; LN, lymph node; R2, second relapse; R3, third relapse; R4, fourth relapse

**FIGURE 3:**
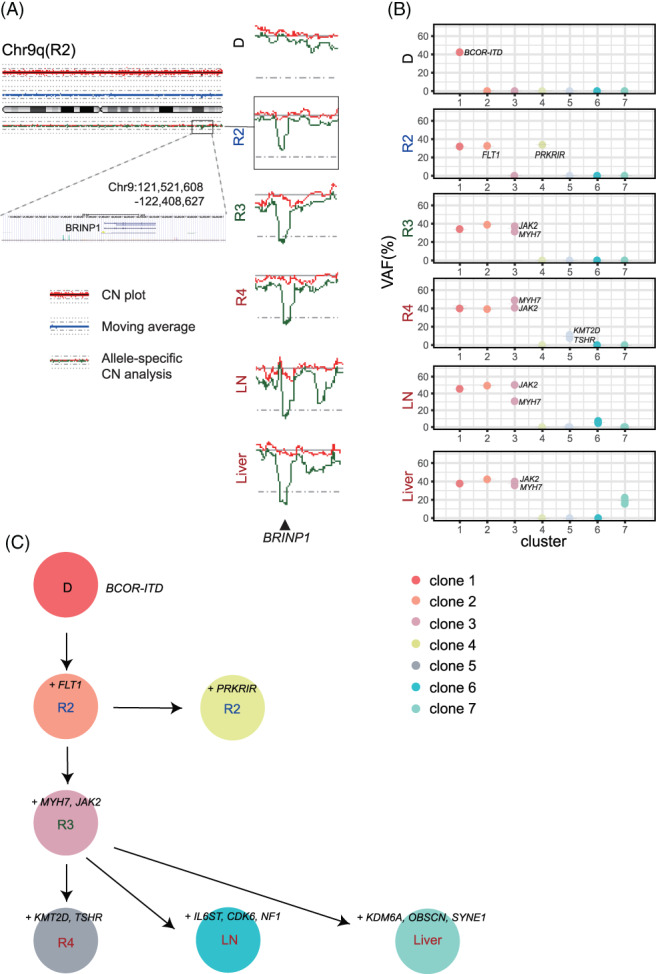
(A) The results of copy number analysis of SNP array for chromosome 9. Red dots at the top represent the signal from each probe (raw data), and blue line indicates the moving average of red dots. Red and green lines at the bottom represent allele‐specific copy number. Allelic loss of chromosome 9q including *BRINP1* gene was observed in R2, R3, R4, LN, and liver samples. (B) Each mutation detected by TCS was classified into seven clones according to its variant allele frequency (VAF). VAFs of mutations and estimated cell population of each sample are shown. (C) The schematic representation of clonal evolution constructed from somatic mutations detected by TCS. *BCOR*‐ITD was the only truncal mutation detected in both primary and relapse samples. Additional mutations accumulated during disease progression were shown. CN, copy number; D, initial diagnosis; LN, lymph node; R2, second relapse; R3, third relapse; R4, fourth relapse; VAF, variant allele frequency

After obtaining informed consent from his parents, DNA was extracted from the peripheral blood and biopsy samples obtained at initial diagnosis, each relapse (second relapse; R2, third relapse; R3, fourth relapse; R4) and autopsy (abdominal lymph node; LN, liver; Liver) except for the sample at first relapse. Direct sequencing identified *BCOR*‐ITD (c.5526_5621dup) at all the analyzed samples (Figure S2). SNP array analysis did not reveal any copy number alterations (CNAs) at the diagnosis whereas consistent CNAs on chromosomes 1q, 7q, and 15q were detected in R2 through R4 as well as LN and liver (Figure 2). Each sample possessed several sample specific CNAs. Moreover, we detected a focal loss at chromosome 9q32‐33 in the samples of R2 through autopsy, where only the tumor suppressor gene *BRINP1* was located (Figure 3A). We further analyzed other genetic alterations using targeted‐capture sequencing (TCS) for 381 genes relating to pediatric solid tumors.^12^ Somatic mutations were filtered using the peripheral blood sample as germline control. Candidate somatic mutations located in exonic regions were further filtered by excluding variants: (a) with a VAF < 0.04; (b) listed in SNP databases; and (c) synonymous single‐nucleotide variants, according to our previous report.^12^ Finally, 13 mutations were detected and classified into 7 clones based on the detected samples and VAF (Table 1 and Figure 3B). Although validation of the candidate mutations was not performed in this study, our sequence depth (mean 600‐714) combined with EB call (Empirical Bayesian Mutation calling) analysis^13^ in Genomon software (https://github.com/Genomon-Project/GenomonPipeline) was estimated to be sufficient to rule out sequencing artifacts. Only the *BCOR*‐ITD was included in clone 1, whereas three clade mutations (*FLT1*, *JAK2*, and *MYH7*) were additionally detected in relapsed tumor samples (clones 2, 3). The other mutations (*PRKRIR*, *KMT2D*, *TSHR*, *CDK6*, *IL6ST*, *NF1*, *KDM6A*, *OBSCN*, and *SYNE1*) were clone specific (clones 4‐7). The number of detected mutations increased during each tumor recurrence. All R3 mutations (clones 1‐3) were detected upon metastatic samples from autopsy, suggesting the accumulation of metastasis site‐specific mutations during tumor progression. Importantly, the newly acquired mutations at each relapse were not detected even as a minor clone in the previous samples, though variants with a VAF < 0.02 were not detectable due to our mutation calling method (Supplementary Material S2). We analyzed the clonal evolution of the present case based on the VAF of each mutation and constructed a node‐based phylogenetic tree using a bootstrap resampling technique implemented in ClonEvol.^14^, ^15^ Longitudinal sequencing analysis at different time points allowed us to delineate the clonal history of tumor cells, suggesting that each relapse developed based on the preexisting tumors (Figure 3C).

**TABLE 1:** Variant allele frequency of each mutation

Clone	Gene	Amino‐acid change	Variant allele frequency (%)					
			D	R2	R3	R4	LN	Liver
1	*BCOR*	L1690_1721Ddup	42	32	34	40	46	38
2	*FLT1*	M148I	—	33	39	39	49	42
3	*JAK2*	L892V	—	—	37	41	50	40
3	*MYH7*	K542R	—	—	31	49	31	36
4	*PRKRIR*	E158Q	—	34	—	—	—	—
5	*KMT2D*	D3165fs	—	—	—	11	—	—
5	*TSHR*	P3fs	—	—	—	8	—	—
6	*CDK6*	K93Q	—	—	—	—	5	—
6	*IL6ST*	T178A	—	—	—	—	6	—
6	*NF1*	V2046L	—	—	—	—	7	—
7	*KDM6A*	Q230L	—	—	—	—	—	23
7	*OBSCN*	R8618Q	—	—	—	—	—	13
7	*SYNE1*	A7572E	—	—	—	—	—	18

Abbreviations: D, initial diagnosis; dup, duplication; fs, frameshift.

## 4 DISCUSSION

In this study, we presented a case of CCSK, which underwent four relapses. The first relapse occurred in the lung. This is consistent with the previous reports that the lung was the most common site of relapse in patients treated with DD‐4A,^4^, ^5^ although the present case relapsed earlier (4 vs 24 months).^1^, ^3^ The optimal treatment of relapsed CCSK has not been established as yet. ICE chemotherapy, a salvage regimen for recurrent CCSK,^6^, ^16^ resulted in the achievement of the second remission, however, the second relapse developed. Due to the refractory disease, we performed high dose chemotherapy with aHSCT rescue though its effectiveness is unclear.^6^, ^16^ Pathological analysis of post‐treatment specimens revealed a partial response, consistent with clinical therapeutic resistance. Because treatment options for relapsed CCSK are limited and survival is poor, we retrospectively analyzed this case in terms of genomics for the possibility of new therapeutic strategies.

In this study, the same *BCOR*‐ITD alteration was observed from diagnosis right up to autopsy with very low tumor mutation burden, suggesting a strong impact not only on CCSK tumorigenesis as previously reported^7^, ^8^ but also on its relapse. Meanwhile, we could not observe truncal mutations that can be targetable with available FDA‐approved drugs.

Accumulation of the genetic abnormalities during tumor progression might be associated with the therapeutic resistance in this case. *BRINP1*, lost in the samples of R2 through autopsy, is a tumor suppressor gene, which regulates the G1/S checkpoint and cell cycle^17^ and its dysregulation is associated with tumor recurrence in other carcinomas.^18^ Our clonal analysis of the metastatic samples suggested that relapsed tumors were derived from minor clones of the preceding tumors, which had survived chemoradiotherapy and thrived (Figure 3C). Moreover, multisampled extractions at autopsy revealed site‐specific mutations of metastasis in addition to truncal mutations of R3 sample, which suggests the presence of subclones with site‐specific mutations in each metastases site of the previous samples. Among them, a subclone with selective advantage might relapse as the dominant clone. This is supported by the result that the newly acquired mutations were not detected even as a minor clone in the previous primary samples. However, our study is based on a single case and functions of the newly acquired genetic alterations remain unclear. In addition, our analysis in the present study was limited to genomic abnormalities, not including other various factors such as epigenetic abnormalities and tumor microenvironment. Further studies with more samples will help to detect common mechanisms of relapse and therapy resistance in CCSK, enabling appropriate therapy for relapsed cases considering genetic status.

Altogether, we failed to find targetable lesions, while our study indicated the importance of *BCOR*‐ITD in both CCSK tumorigenesis and relapse. The genetic alterations acquired during disease progression might contribute to clonal selection and therapeutic resistance in the present case though further studies with more samples are warranted.

## CONFLICT OF INTEREST

The authors declare no conflicts of interest.

## AUTHOR CONTRIBUTIONS

**Tomoki Yaguchi:** Data curation; formal analysis; investigation; visualization; writing‐original draft; writing‐review & editing. **Shunsuke Kimura:** Data curation; formal analysis; investigation; visualization; writing‐original draft; writing‐review & editing. **Masahiro Sekiguchi:** Data curation; formal analysis; investigation; visualization; writing‐original draft; writing‐review & editing. **Yasuo Kubota:** Investigation. **Masafumi Seki:** Data curation; formal analysis; investigation; visualization; writing‐original draft. **Kenichi Yoshida:** Investigation. **Yuichi Shiraishi:** Investigation. **Keisuke Kataoka:** Investigation. **Yoichi Fujii:** Investigation. **Kentaro Watanabe:** Investigation. **Mitsuteru Hiwatari:** Investigation. **Satoru Miyano:** Investigation. **Seishi Ogawa:** Conceptualization; funding acquisition. **Junko Takita:** Conceptualization; funding acquisition; project administration; supervision; visualization; writing‐original draft; writing‐review & editing.

## ETHICAL STATEMENT

The present study was approved by the Ethics Committee of the University of Tokyo. Informed consent for publication was obtained from the patient's parents.

## Supporting information


**Figure S1**. Computed tomography imaging during the clinical course of the present case, A; at initial diagnosis, B; at first relapse, C; at second relapse, D; at third relapse, E, F, G, H, and I; at fourth relapse. White arrows represent tumor site.

**Figure S2**. A; *BCOR*‐ITD (c.5296_5391dup; NM_0177455) was detected from all tumor samples except for R1. The reference sequence (top) and the sequence data obtained from primary sample (bottom) are shown. The parental (red) and duplicated (blue) segments are indicated.
B; Genomic PCR analysis of *BCOR* exon 15. The PCR products from samples in D, R1, R2, R3, R4, LN, and Liver are presented. Targeted PCR and gel electrophoresis of *BCOR* exon 15 in samples from D, R2, R3, LN and Liver showed large product. C; Schema representing structural variant of *BCOR*‐ITD. Parental segment in PUFD domain was duplicated (ITD). M, marker: C, control (peripheral blood); D, initial diagnosis; R2, second relapse; R3, third relapse; R4, fourth relapse; LN, lymph node; ANK, ankyrin repeats; PUFD, PCGF ubiquitin‐like fold discriminator.

## Data Availability

The data that support the findings of this study are available on request from the corresponding author. The data are not publicly available due to privacy or ethical restrictions.
