# Supplementary material for: Description of longitudinal tumor evolution in a case of multiply relapsed clear cell sarcoma of the kidney
Source: Cancer Rep (Hoboken). 2021 Dec 29;5(2):e1458. doi: 10.1002/cnr2.1458 (PMC8842696; doi:10.1002/cnr2.1458)
Supplement: Supplementary file 1 — Figure S1. Computed tomography imaging during the clinical course of the present case, A; at initial diagnosis, B; at first relapse, C; at second relapse, D; at third relapse, E, F, G, H, and I; at fourth relapse. White arrows represent tumor site. [file CNR2-5-e1458-s001.docx]

**Supplementary Material S1**

**Pathological findings**

**Second relapse**

Histologically, it is consistent with clear cell sarcoma with treatment-induced degeneration. There is a proliferation of round or spindle-shaped cells, but in general the cell density is low. Fibrous or mucous matrix formation is prominent, along with hemorrhage, hemosiderin deposition, foam cell aggregation, calcification, infiltration of inflammatory cells (mainly lymphocytes), and proliferation of small blood vessels.

**Third relapse**

Histologically, the lesion consists of necrotic tissue, fibrotic tissue, and granulation tissue, with residual degenerated tumor. Distributed tumor cells have round nuclei with well-defined nucleoli and pale eosinophilic cytoplasm. Post-tumor changes such as myxoid degeneration, fibrosis, hemorrhage, and hemosiderin deposition are prominent.
