# Supplementary material for: Description of longitudinal tumor evolution in a case of multiply relapsed clear cell sarcoma of the kidney
Source: Cancer Rep (Hoboken). 2021 Dec 29;5(2):e1458. doi: 10.1002/cnr2.1458 (PMC8842696; doi:10.1002/cnr2.1458)
Supplement: Supplementary file 2 — Figure S2. A; BCOR‐ITD (c.5296_5391dup; NM_0177455) was detected from all tumor samples except for R1. The reference sequence (top) and the sequence data obtained from primary sample (bottom) are shown. The parental (red) and duplicated (blue) segments are indicated. B; Genomic PCR analysis of BCOR exon 15. The PCR products from samples in D, R1, R2, R3, R4, LN, and Liver are presented. Targeted PCR and gel electrophoresis of BCOR exon 15 in samples from D, R2, R3, LN and Liver showed large product. C; Schema representing structural variant of BCOR‐ITD. Parental segment in PUFD domain was duplicated (ITD). M, marker: C, control (peripheral blood); D, initial diagnosis; R2, second relapse; R3, third relapse; R4, fourth relapse; LN, lymph node; ANK, ankyrin repeats; PUFD, PCGF ubiquitin‐like fold discriminator. [file CNR2-5-e1458-s002.docx]

**Supplementary Material S2**

**Material and Methods**

DNA was extracted from the peripheral blood and biopsy samples at initial diagnosis (D), each relapse (second relapse; R2, third relapse; R3, forth relapse; R4) and autopsy (abdominal lymph node; LN, liver; Liver) except for the sample at first relapse after obtaining informed consent from his parents following protocols of the Human Genome, Gene Analysis Research Ethics Committee of the University of Tokyo. Direct sequencing of *BCOR* was performed according to manufacturer's protocol with the use of an ABI 3500 Genetic Analyzer (Applied Biosystems; Thermo Fisher Scientific, Inc., Waltham, MA, USA). The following set of primers were used: BCOR_F: GACACATATGCACAAGGATTAACAC; BCOR_R: TGGGCGCACTTTTCATTT. Genomic assay of DNA extracted from biopsied samples was performed by SNP array analysis with the Affymetrix GeneChip 250K Nsp system (Affymetrix) following the manufacturer’s protocol. CNAG/AsCNAR software was used for analysis of the SNP array data^1^. Targeted-capture sequencing (TCS) was performed using a SureSelect custom kit (Agilent Technologies). Our custom bait library (U-Tokyo Onco-panel ver.1) included 381 targeted genes and regions. Sequence alignment and detection of gene mutations and structural variations were performed using our in-house pipeline, Genomon v2.5.2 (<https://github.com/Genomon-Project/GenomonPipeline>) with the following the parameters: (i) mapping quality score ≥20; (ii) base quality score ≥15; and (iii) VAFs in tumor samples ≥0.02. Somatic mutations were filtered using the peripheral blood sample as germline control. Candidate somatic mutations located in exonic regions were further filtered by excluding variants: (i) with a VAF <0.04; (ii) with an EBcall^2^ (Empirical Bayesian mutation calling) P >1 ×10^−4^; (iii) with a Fisher exact P >1 ×10^−4^; (iv) detected on only either the plus or minus strand; (v) listed in the NCBI dbSNP build 131, the Human Genome Variation Database (HGVD; April 2016 release), or our in-house SNP database; and (vi) synonymous single-nucleotide variants. Mean sequencing depths were 713 (D), 713 (R2), 600 (R3), 606 (R4), 699 (LN), and 714 (Liver), respectively.

**References for Supplement**

1. Nannya Y, Sanada M, Nakazaki K, et al. A robust algorithm for copy number detection using high-density oligonucleotide single nucleotide polymorphism genotyping arrays. Cancer Res. 2005;65(14):6071-6079.

2. Yuichi Shiraishi, et al. An empirical Bayesian framework for somatic mutation detection from cancer genome sequencing data. Nucleic Acids Res 2013;41:e89.
